# Meaning of Aging, Gerotranscendence, and Successful Aging in Older Adults With Chronic Health Conditions

**DOI:** 10.1093/geroni/igaa057.1364

**Published:** 2020-12-16

**Authors:** Meredith Troutman-Jordan

**Affiliations:** University of North Carolina, Charlotte, North Carolina, United States

## Abstract

Aging presents change in the form of opportunities and challenges, from common physical alterations, to major life events. Perception of such events is greatly shaped by one’s mental health, and is a major influence on gerotranscendence, a positive kind of aging involving redefinition of self, relationships, and proposed to be a precursor to successful aging. A mixed-methods cross-sectional descriptive design was used to study 50 older adults. Life Events Checklist, Gerotranscendence Scale, Herth Hope Index, and Successful Aging Inventory were administered. Mean participant age was 70.78 years; there were 9 males (18%), 41 females (82%), 13 were Black (26%), and 37 were White (74%). Participants reported a number of stressful events, most frequently transportation accidents, followed by other very stressful events or experiences, and sudden unexpected death of someone close. Gerotranscendence scores ranged from 0-10 (µ 6.88, a moderate score). Successful aging scores ranged from 40-79 (µ 62.33, a moderate score). A sub-sample of 6 participants engaged in semi-structured interviews, which were transcribed verbatim and subject to content analysis. Faith, displaced longing, temporal anticipation, proactive problem-solving/coping, and concern for future generations were emergent qualitative themes. Findings highlight opportunities for providers from multiple disciplines to target risks and possibilities for aging successfully and to promote hope, optimism, problem-solving skills, and gerotranscendence in all older adults, regardless of physical or functional health status.

